# Œdème aigu hémorragique du nourisson

**DOI:** 10.11604/pamj.2019.33.191.16154

**Published:** 2019-07-12

**Authors:** Fatima-Zahra Agharbi

**Affiliations:** 1 Hôpital Civil Tétouan, Tétouan, Maroc

**Keywords:** Œdème, aigu, hémorragique, nourrisson, pseudo cocardes, Edema, acute, hemorrhagic, infant, pseudoannular

## Abstract

In infants less than 2 years of age, acute hemorrhagic edema of infancy (AHEI) is characterized by the rapid onset of annular purpuric lesions associated with initially localized edemas affecting the extremities. This disorder is usually benign, without visceral involvement. Diagnosis is based on clinical examination, no specific laboratory findings exist and the histological examination of the lesions (which is unnecessary in the majority of cases) is most often nonspecific (sometimes patients have nonspecific leukocytoclastic vasculitis). Nosological status of AHEI remains unclear, but some reports suggest it is a clinical manifestation of rheumatoid purpura. Rhinopharyngeal episodes in the days before its onset have sometimes been reported, suggesting a viral cause. There is a net mismatch between patient's good general condition and the remarkable dissemination of the lesions. Infants monitoring must be rigorous in the first days, even though complications are exceptional (acute intussusception). Patients recover spontaneously within 12 days. Therapeutic management is based on accurate monitoring of the infant's general status. Fever, the extension of the purpuric lesions and, in particular, signs of impaired general condition may suggest the diagnosis of purpura fulminans. Another differential diagnosis is acute hemorrhagic or ecchymotic urticaria. We here report the case of a 3-month old infant with diffuse pseudoannular lesions within a context of apyrexy and preservation of patient's general condition. Patient's outcome was favorable without any treatment, confirming the diagnosis of AHEI.

## Image en médecine

L'œdème aigu hémorragique du nourrisson (OAHN) est caractérisé par la survenue rapide de lésions purpuriques en cocardes, associées à des œdèmes initialement localisés aux extrémités chez l'enfant de moins de 2 ans. Cette affection reste le plus souvent bénigne, sans atteinte viscérale et le diagnostic est clinique, il n'existe pas de signes biologiques spécifiques et l'analyse histologique des lésions (qui est inutile dans la majorité des cas) est le plus souvent sans spécificité (il existe parfois une vascularite leucocytoclasique non spécifique). Sa place nosologique reste incertaine, faisant discuter, pour certains, une forme clinique du purpura rhumatoïde. On retrouve parfois un épisode rhino-pharyngé dans jours précédents faisant discuter une étiologie virale. Il existe une discordance nette entre le bon état général et l'aspect profus et spectaculaires des lésions. La surveillance de ces enfants dans les premiers jours doit être rigoureuse mais les complications sont exceptionnelles (invagination intestinale aiguë?). L'évolution se fait spontanément vers la régression en une dizaine de jours. La prise en charge thérapeutique consiste en une surveillance attentive de l'état général de l'enfant. Une fièvre, l'extension des lésions purpuriques et surtout des signes d'altération de l'état général peuvent faire discuter le diagnostic de purpura fulminans. Un autre diagnostic différentiel est l'urticaire aigue hémorragique ou ecchymotique. Nous rapportons l'observation d'un nourrisson de 3 mois qui présente des lésions pseudo-cocardes diffuses dans un contexte d'apyrexie et de conservation de l'état général. L'évolution était favorable sans aucun traitement ce qui a permis de confirmer le diagnostic d'OAHN.

**Figure 1:**
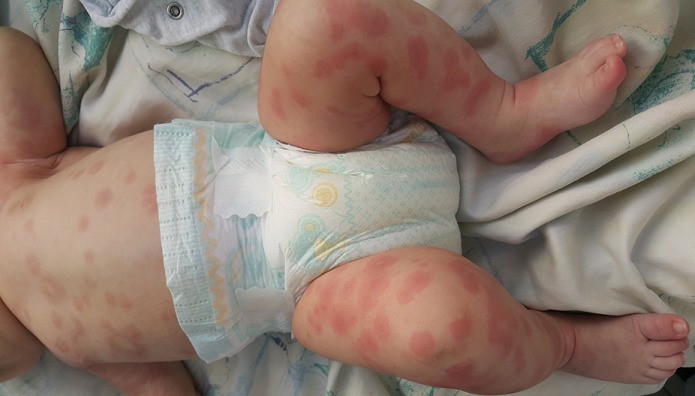
Lésions pseudo-cocardes diffuses

